# Treatment of liver and spleen illnesses by herbs: Recommendations of Avicenna’s heritage "Canon of Medicine" 

**Published:** 2019

**Authors:** Mozhgan Ghobadi Pour, Naser Mirazi, Asghar Seif

**Affiliations:** 1 *Department of Biology, Faculty of Basic Sciences, Bu- Ali Sina University,* *Hamedan, Iran*; 2 *Department of Statistics, Faculty of Basic Sciences, Bu- Ali Sina University,* *Hamedan, Iran*

**Keywords:** Herbal medicine, Liver, Materia medica, Spleen

## Abstract

**Objective:**

Avicenna (Abu Ali al-Hossein ibn Abdullah ibn Sina) who had a special attention toward diseases treatments, gathered results of ages of herbal medicine experiments on humans and animals in his book “Al-Qānūn fī Ṭibb” or "The Canon of Medicine", which is a reliable book in Iranian traditional medicine.

The aim of this research was to build a reliable list of plants effective against liver and spleen diseases, based on Avicenna's book (volume 2).

**Materials and Methods:**

By studying the monographs, introduced agents that have been effective in liver and spleen diseases were identified. Upon their origin and effectiveness in diseases of the liver, spleen or both, treatments were organized.

**Results:**

From a huge number of drugs, 163 plants from 73 families were found to be effective in treatment of liver and spleen illnesses. In addition, 30 non-herbal agents effective in treatment of liver diseases were detected. The Lamiaceae family have the most effective herbs for treatment of diseases of the liver, spleen or both. Hemp Agrimony, Irsā, and Fūdhanj achieved the highest scores.

**Conclusion:**

The effects of different plants on liver and spleen diseases were indicated in Avicenna's book. Due to the report on the above book, further studies needed specially on the effect of Irsā (Iris ensata) and family Lamiaceae on liver and spleen diseases.

## Introduction

The largest organ in the body is the liver, comprising about 2 percent of the total body weight; in an adult with average body mass, the liver is about 1.5 kg. The liver performs many different functions including: 1) filtration and storage of blood; 2) metabolism of carbohydrates, proteins, fats, hormones, and foreign chemicals; 3) formation of bile; 4) storage of vitamins and iron; and 5) formation of coagulant factors (Hall, 2015 ▶). Liver diseases are conditions that affect the liver. The liver is prone to diseases due to multidimensional functions and its location (Kumar et al., 2014 ▶). The efficiency of current synthetic agents in treating chronic liver disease is not satisfactory and these chemicals have undesirable side effects. Thereby, numerous phytochemicals and medicinal herbs, as alternative and complementary treatments, have been investigated for chronic liver diseases (Hong et al., 2015 ▶). Iranian traditional medicine (ITM) has been used for prevention, diagnosis, and treatment of diseases and this medicine works based on the humor theory of temperament in which, the liver is one of the most important organs in the body (Akbarzadeh et al., 2015 ▶).

Al-Hossein Abu-Ali Ben Abdullah, Ibn Sina, (known as Sheikh al-Rais (or the Prince of the physicians) and in the West as Avicenna, 980-1037 AD) was an extremely talented individual. Avicenna practiced philosophy, astronomy, geometry, mathematics, and medicine as well as poetry and music. Although medicine was not his main area of interest, he became famous as a physician due to the desperate need for thoughtful medical personnel in the Persian kingdom. Among Avicenna’s works, his medical book *Al-Qānūn fī Ṭibb, *known as *Canon *in the Western Hemisphere, has a great scientific and historical value. *Canon *is written in three parts. Part I covers the anatomy and physiology of the human body; Part II includes the description, signs, and symptoms of the disease and Part III describes the treatment of disease and prophylactic measures to prevent disease. For treatment of most diseases, he used food, psychotherapy , and medicinal plants (Qayumi, 1998 ▶). Arturo Castiglioni appreciated Avicenna's Canon: "The clarity of the clinical histories, the accuracy of the therapeutic indications, constructed logically and without dangerous exaggerations, and the eloquence of his forcible style were sufficient to confer on this book up to the end of the seventeenth century an almost indisputable authority in the minds of the physicians of all countries" (Galdston, 1955 ▶).

Acute liver disease damages the spleen in long term. In Book 2 (the *Materia Medica*) of Canon, Avicenna alphabetically listed about 806 simple medical agents (of floral, mineral, and animal origin) that were used at the time. Each agent may have different possible general actions, followed by specific properties listed according to symptoms of liver and spleen diseases. In floral monographs of Canon, we found that Avicenna has found that some herbs cure some symptoms so we considered such symptoms and searched for herbs with such properties.

## Materials and Methods

**Figure 1:**
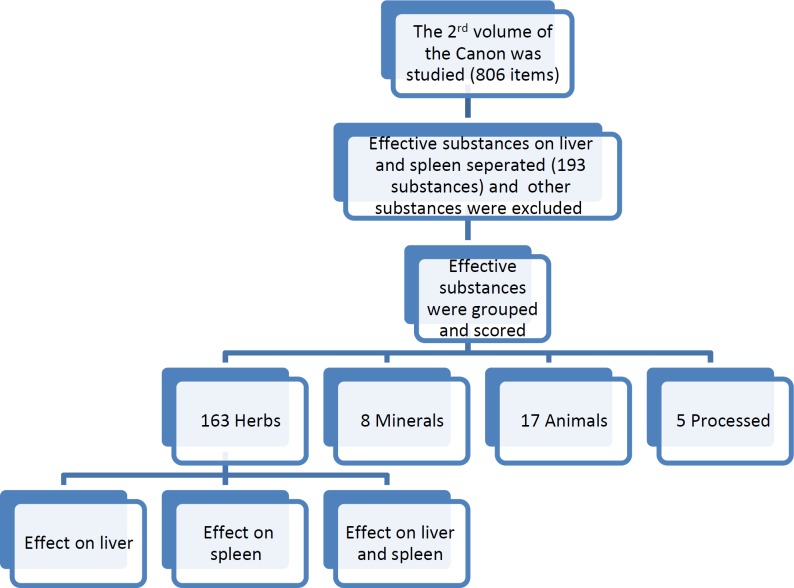
The flow chart of the study

In our evaluation, we used different versions of the Canon book available at: (https://sites.google.com/site/avicennacanon1a/canon-web-htm). This library was created by Dr. Hossein Hatami and is also accessible through the Bu Ali Sina Scientific and Cultural Foundation website (http://www.buali.ir/). The following versions of the Canon were used in our study: 1) The corrected version of Canon in Persian (Sina, 2010 ▶), 2) Arabic manuscript of the Canon (Ibn Sina, 2005 ▶), and 3) Translated version of the Canon in English (Sīnā, 1998 ▶). As the first step, to indicate which herbs have hepatoprotective and other effects for liver diseases, the 2nd volume of Canon was searched. These items were mutually compared and evaluated. Subsequently, data were collected based on different plant species in the areas of healing, and protection. The flow chart of the study is presented in Figure 1.

**Statistical analysis**

The average values for results are expressed as a mean± standard error of mean (SEM). Statistical analysis was performed using the Statistical Package for the Social Science (IBM SPSS statistics version 23) program for Windows. Statistical significance of differences between groups was evaluated using non-parametric statistics, the relationship between the numbers of herbs in each family to score of herbs was shown by the Kruskal–Wallis test p<0.05 was considered statistically significant. Graphs were created with Excel 2013 software (Microsoft office 2013).

## Results

Avicenna introduced 193 agents as they were effective on the liver and spleen. Their characteristics are presented in four categories according to the origin of agent in Tables 1-6 which present basic information such as common name, Persian name, Arabic name, scientific name, family, used parts (i.e. root, fruit, etc.)/ mode of consumption (fried, roasted, etc.) or preparation (enema, smell, etc.), diseases for which the agent was prescribed and finally score.

**Figure 2:**
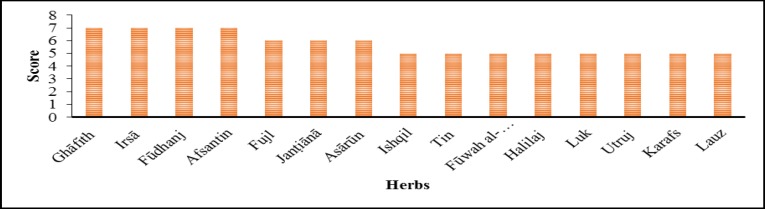
. Effective herbs against liver and spleen diseases mentioned in Avicenna’s book. Scores are according to the number of effects that every herb has been prescribed by Avicenna

**Figure 3:**
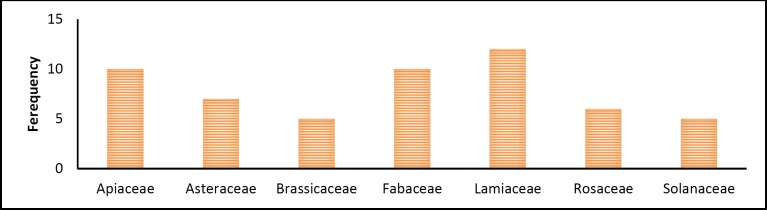
Histogram of families with the highest number of effective herbs

**Figure 4:**
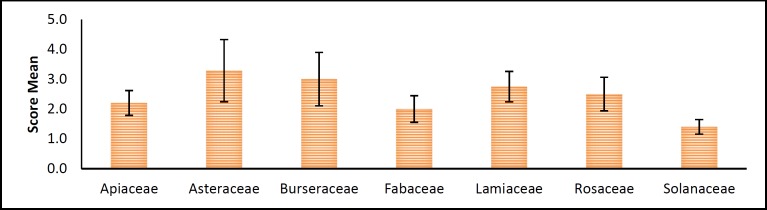
Relationship between the plant families and a mean of scores of their herbs. Data are presented as mean ± SEM. p>0.05

**Plants**

We reviewed all herbs mentioned in the 2^nd^ volume of Canon and found a total of 163 plants used for liver and spleen illnesses. These plants belong to 73 different families. These herbs included medicinal, poisonous, ornamental and economic plants and weeds that are presented in Tables 1-3. According to their effectiveness, they are categorized as effective on liver Table 1, spleen Table 2 and both liver and spleen Table 3. The second volume of the canon book consists of a series of monographs each one describing different properties of one medicinal herb. The monograph name that described the properties of No. 92 herb is lost during repeated transcription through ages so it has indicated as "???" in Table 1. Herb number 93 is a type of endive with no scientific name.

**Table 1:** Data from Canon book 2 about herbs with hepatoprotective/hepatotherapeutic effects

***No.***	***Persian Name***	***Common name***	***Arabic name***	***Scientific Name***	***Family***	***Used Parts/*** **Mode of con*sumption *or preparation**	***Conditions which the herb has effect on***	***Score***
***1***	Piyāz	Onion	Baṣi	*Allium cepa*	Amaryllidaceae	/ Twice fried (or roasted)	J	1
***2***	Sir	Garlic	Thūm	*Allium sativum *L.	Amaryllidaceae	/ Enema.	BH	1
***3***	Peste	Pistachio nut	Fustuq	*Pistacia vera *L.	Anacardiaceae	/ Smell, oil, syrian variety	OL, CLL, PL	3
***4***	Somāq	Sumach	Summāq	*Rhus coriaria *L.	Anacardiaceae	/ Pickle	PF, BN	2
***5***	Nane havvā	Ajowan	Nānkhāh	*Carum copticum *L.	Apiaceae		CL	1
***6***	Zire biyābāni	Cumin	Kammūn	*Cuminum cyminum *L.	Apiaceae		FD	1
***7***	Šire-ye- 'angodān, salqiun	Asafoetida	Ḥiltit	*Ferula assafoetida *L.	Apiaceae	/ Ingestion	J	1
***8***	Sakbine	Sagapanum	Sakbinaj	*Ferula persica *Willd*. *	Apiaceae	/ Poltice	D, RY, PL	3
***9***	Sefandufalaiun, safanduliun	Wild cumin	Saqandūliūn, safandūliūn, safidūliūn	*Heracleum spondylium *L*.*	Apiaceae	Root	PL, J	2
***10***	Gaz[']angabin e estabrak'	Sweet exudate of saccharum	Sukkar al-'ushr, 'ushr	*Calotropis procera *R.Br*.*	Apocynaceae	/ Ingestion	D, GL	2
***11***	Mārčube, miyān asfārāghas, mawāqinūs	Hilyun	Hilyūn, mevaqensūs, zaqyūs	*Asparagus officinalis *L*. *	Asparagaceae	Root, seed / cooked	OL, J	2
***12***	Alvā'	Small aloa	Sibr	*Aloe littoralis *	Asphodelaceae	/ Oral intake	HBF, OL, J, EXB	4
***13***	Xonsā	Asphodel	Khuntha	*Asphodelus tenuifolius * *Asphodelus *sp*.*	Asphodelaceae		J	1
***14***	Kāsni	Endive, chicory	Hindabāʾ	*Cichorium intybus *L*.*	Asteraceae	/ Bibtter variety	OL, UL, EXC, LT,	4
***15***	Kāhu	Lettuce	Khas	*Lactuca sativa *L*.*	Asteraceae	/ With vinegar	J	1
***16***	Bābune	Chamomile	Bābūnaj	*Matricaria chamomilla *L*.*	Asteraceae		J	1
***17***	Kangare xar, čarxe	Arabian thorn, multi-knotted	Shukā'i, kathir al-ͨaqd	*Onopordon arabicum *L*.*	Asteraceae		UL	1
***18***	Gušfil	Arum	Ghalghaas, ghalghassh	*Colocasia antiquorum *Schott & Endl.	Araceae		BD	1
***19***	Zerešk	Barberry	Amberbāris	*Berberis aristata*	Berberidaceae		EYB, STL	2
***20***	'Afiyus, 'Afnus	Horse radish root	Afiyūs, Afnūs	*Raphanus agria*	Brassicaceae	Fruit	J	1
***21***	Bašām	Balm of gilead balsam	Balsān, balsān makki	*Commiphora opobalsamum *(L.) Engl.	Burseraceae	/ Cooked	STL	1
***22***	Nārmošk	Iron wood tree	Nārmushk, nāghisht	*Mesua ferrea *L*.*	Calophyllacea		CL	1
***23***	Šāhdāne	Shahdanaj, hemp seed	Shahdānaj	*Cannabis sativa *L*.*	Cannabaceae	/ Juice= shāhdānaq	EXB	1
***24***	Esfe(a)nāj'	Spinach	Asfānākh	*Spinacia oleracea *L*.*	Chenopodiaceae		CB	1
***25***	Mahmude	Scammony	Saqmūniā	*Convoluvulus camononia* *Convolvulus scammonia *L*. *	Convolvulaceae	Root / oral intake	PB	1
***26***	Košus	Dodder	Kashūth	*Cuscuta reflexa*	Convolvulaceae	/ Orally, juice	OL, STL, J	3
***27***	Motā'	Dogwood	Mū	*Cornus mascula*	Cornaceae		CL, GIL	2
***28***	Šarang, hendevāne-ye abujahl'	Colocynth	Ḥanẓal	*Citrullus colocynthis *L*.*	Cucurbitaceae	Root	D	1
***29***	Simāhang, xiyār e 'olāq	Squirting cucumber	Qiththā' al-ḥimār	*Momordica elaterium *L*.*	Cucurbitaceae	Roots, leaves, barks / extract, orally, decocted	J, D, VB, EVY	4
***30***	Šarhi, avers'	Sharbin, cedar tree	Sharbin	*Chamaecyparis *sp*.*	Cupressaceae	Fruits	UL	1
***31***	Mo(e)šk e za(e)min	Indian cypress	S'ad	*Cypress rotundus *L*.*	Cyperaceae		PH, D	2
***32***	Dome asb,' shenge čamani	Horse tail	Dhanab al-khail	*Equisetum arvense *L*.*	Equisetaceae		IL, D	2
***33***	Māhudāne	Caper-spurg, myrtle spurge, wild caper	Māhudānah, hab[b]-bol-moluk, al-sisbān	*Croton tiglium *L*. *	Euphorbiaceae	Seed	D, PB	2
***34***	Gāvkašk	Spurge	Shabram	*Euphoarbia pithyusa*	Euphorbiaceae	/ Orally, soaked	D	1
***35***	Karčak	Castor	Kheroo, qarāvatia	*Ricinus communis *L*.*	Euphorbiaceae	Seed/ attrited	EXB	1
***36***	Rame	Soap nut	Rittah	*Caesalpinia bonduc *(L.) Roxb. *Syn, Guilandina bonduc*	Fabaceae	/ Scuash	EBB, EVY, J, I	4
***37***	Xiyaršambar	Purging cassia	Khiār shambar	*Cassia fistula *L	Fabaceae		CLL, J, PL, EVY	4
***38***	Xarnub	Ceratonia carob, nabatean carob, yanbūt	Kharnūb	*Ceratonia siliqua *L*.*	Fabaceae	Yanbūt	J	1
***39***	Taranja(e)bin, hāj, xāršotor	Manna	Taranjubin, alhāji- maurorum, khare āqul',	*Hedysarum alhagi *Lerche*.*	Fabaceae		PB	1
***40***	Tamre hendi	Tamarind	Tamr hindi	*Tamarindus indica *L*.*	Fabaceae	/ Decoction	PB	1
***41***	Ney e nahāvandi, ney e zarire	Chiratta	Qaṣab al-dharirah, dharirah	*Swertia chirata *(Wall.) C.B. Clarke.	Gentinaceae		IL, D	2
***42***	Lāk	Lac	Luk	*Coccus lacca* *Syn Kerria lacca* *Ficus laccifera *Roxb*. * *Cataris lacca *	Kerriidae		UL, STL, J, D, PL	5
***43***	Na'nā'	Spicata spearmint	Na ͨnȧ ͨ	*Mentha sativa *L*.*	Lamiaceae	/ Water	J	1
***44***	Sangol, zufā ye tar		Zūfā ratb	*Nepta orientalis *Mill*.*	Lamiaceae	/ Painted or taken orally	CL, D	2
***45***	Sumarn, toxm e zardāb		Thūmūn	*Thymus capitatus *LK&H.	Lamiaceae	/ Syrup	BH	1
***46***	Pune koohi, marze ye koohi, marze	Origanum	Sa'tar	*Zataria multiflora*	Lamiaceae		LD	1
***47***	Dārčin khataei	Cassia bark	Salikhah	*Cinnamomum cassia *auct*.* *Syn, Cinnamomum aromaticum*	Lauraceae	Bark / syrup, infusion	UL	1
***48***	Dārčin	Cinnamon	Dār ṣini	*Cinnamomum zeilanicum Blume *var. cassia Nees *Syn. Cinnamomum verum*	Lauraceae		OL, STL, D	3
***49***	Anār'	Pomegranate, carthaginian apple	Rummān	*Punica granatum *L.	Lythraceae	Seed /sour pomegranate syrup, attrited	CB, BD	2
***50***	Molukiye, panirake bostāni, panirake kāštani	Jews mallow	Mulūkhiā, khubbāzi	*Corchorus olitorius *L.	Malvaceae	Garden variety, Wild variety	OL PB	2
***51***	Garmdāne, gardmāne	Kermes	Karam dānah	*Coccus cacti* *Syn, Protortonia cacti*	Monophlebidae		ED, EXB	2
***52***	Mixak	Cloves	Qaranful	*Caryophyllus aromaticus *L*.* *Syn, Syzygium aromaticum*	Myrtaceae		STL	1
***53***	Murd	Myrtle	Ās	*Myrtus communis *L*.*	Myrtaceae	/ Juice	BD	1
***54***	Zeytun	Oliva	Zaitūn(al-zait)	*Olea europea *L*.*	Oleaceae	/ Paint oil sediment, plaster wild variety	D I	2
***55***	Gol e jāliz	Maltesa mushroom	Ṭarāthith	*Orobanche caryophyllacea *SM *Phelypaea coccinea *Poir	Orobanchacceae		AL	1
***56***	Favina, gol e sad tu(o)māni	Peony	Fāwāniā, ʿŪd al-ṣalib, dhā al-aṣābi' ,'al'isi	*Paeonia officinalis *Retz*.*	Paeoniaceae	Root / orally	J, OL	2
***57***	Xašxāš, šāxdār xašxāš sāheli, qārāltol	Poppy	Khashkhāsh, manqur, khashkhāsh moqarran, khashkhāsh bahri	*Papaver * *Glaucium flavum *Grant	Papaveraceae Papaeraceae	Roots of horned sea poppy / decocted	LD	1
***58***	Zardčube	Turmeric	'urūq al-ṣabbāghin	*Chelidonium majus *L*.*	Papaveraceae		OJ, OL	2
***59***	Šāhtare	Fumitory	Shāhṭarj	*Fumaria officinalis *L*.*	Papaveraceae	/ Orally	OL, I	2
***60***	Vāle	Rock moss	Ushnah	*Peramedia perlata * *Usnea sp. *	Parmeliaceae	/ Soaked in some constipating wine	PL	1
***61***	Tannb	Abies, fir	Tannūb	*Picea abies *(L.) H. Karst.	Pinaceae		LI	1
***62***	Se(a)no[w]bar	Pine, common fir tree	Ṣanobar	*Pinus pinea *L*.*	Pinaceae	Bark and leaves / oral intake	PL	1
***63***	Bārhang, besyār dande ،haft dande	Great plantain, multi angled, seven angled	Lisān al-hamal	*Plantago major *L*.*	Plantaginaceae	Roots, seeds and leaves, extract / a dish of lentil containing great plantain, orally, enemas	OL, D, BD	3
***64***	'Esfarze,'aspiqul	Ispaghola, spogel seed	Bazr qaṭūnā	*Plantago ovata *Forssk*.*	Plantoginaceae	Mucilage	BT	1
***65***	Gur giyāh	Bug rush	Idhkhir	*Andropogon schoenanthus *L*.*	Poaceae	Flowers / oil	I, IL, D	3
***66***	Tabāšir, xeyzarān	Bamboo concretion	Ṭabāshir	*Bambusa arundinacea *Retz*.*	Poaceae	Wood / ash, paint	SIN, BD	2
***67***	Rivās	Ribes	Ribās	*Rheum ribes *L*.*	Polygonaceae		BD	1
***68***	Xorfe	Purslane	Baqla ḥamqā, farfakh.	*Portulaca oleracea *L*.*	Portulacaceae	/ Syrup or paste, enema	RB, IRL, VB, BD	4
***69***	Kabābe	Cubeb	Kabābah	*Cubeba officinalis *Raf*.*	Piperaceae		OL	1
***70***	Marmirān	Golden threat root	Māmirān	*Coptis teeta*	Ranunculacea	Root	J	1
***71***	Xarbaq e siyāh	Black hellebore	Kharbaq aswad, mālinodiol	*Helleborus niger *L*.*	Ranunculaceae		EBB	1
***72***	Zālzālak,	Azarole	Za'rūr, ṭariqāniqūn	*Crataegus melanocarpa *L*. *	Rosaceae		EY, PE	2
***73***	Panj barg, Nitafili	Five leaf grass, cinquefoil	Khamsa aurāq, banṭāfilūn, Niṭāfulūn	*Potentilla reptans *L*.*	Rosaceae	Milk, root / extract	J, PL	2
***74***	Ālu'	Bukhara plum	Ijjāṣ	*Prunus domestica *L*.* *Prunus Spinosa *L*. *	Rosaceae	Old, sweet variety, fresh	EXB	1
***75***	Golābi	Pear	Kummatharā	*Pyrus communis *L*.*	Rosaceae	Chinese variety / rob	REB, BD	2
***76***	Gol e sorx	Rose	Ward	*Rosa damascus * *Rosa damascena *L*. * *Syn, Rosa x damascena *	Rosaceae	Dry flowers / oil	CM, GL, BD	3
***77***	Senjed	Service tree	Ghubairā	*Elaeagnus angustifolius*	Elaeagnaceae		SIN	1
***78***	Utruj, tora(o)nj, bālang	Citron	Utruj, tora(o)nj	*Citrus medica *L*.*	Rutaceae	/ CoIlyrium, juice	RB, J, VB, BD, EJ	5
***79***	Fāxere	Split cubeb	Fāghirā	*Zauthocylum alatum* *Zanthoxylum alatum *Roxb*.*	Rutaceae	/ Incorporated in medicines	CL	1
***80***	Bid e biyābāni	Goat willow	Khilāf	*Salix caprea *L*.*	Salicaceae	Juice	OL, J	2
***81***	Mehrgiyāh	Belladonna	Yabrūh, yabrūj, yabrūh os sanam, moqulen, varqia, riūqes	*Mandragora officinarum *L*. *	Solanaceae	Sap	VB, PB	2
***82***	Kaka(o)ne, arusak'.e pošt.e parde	Winter cherry, alkekeng, bladder	Kākenj	*Physalis alkekengi *L	Solanaceae		J	1
***83***	Bādenjān	Brinjal	Bādhinjān	*Solanum melongena *L*.*	Solanaceae	Cooked with vinegar	OL	1
***84***	Angur'	Garden night shade	ͨinab, ͨinab o s sa'lab	*Solanum nigrum *L*.*	Solanaceae	Resin	UL	1
***85***	sorxdār	Yew	Zarnāb	*Taxus baccata *L*.*	Taxaceae		CL	1
***86***	Aqlaguni, o[w]d'	Eagle-wood, aloe wood	Aghālōgi, aghālōgi, o[w]d' al bo(e)xor , ͨūd	*Aquilaria agallocha, *Roxb *Syn. Aquilaria agallochum*	Thymelaeaceae	Wood / oral intake	PL, STL	2
***87***	Banafše	Sweet voilet	Banafsaj	*Viola odorata *L*.*	Violaceae	/ Dry	EXB	1
***88***	Tāk	Grape vine	Karm	*Vitis vinifera *L*.*	Vitaceae	Root wild grape -vine	D, ED	2
***89***	Hāl bawwā, Khair buwwā	Small cardamom, Lesser cardamom	Hil bawwā, Hāl bawwā, Khair buwwā	*Amomum cardamomum L. Syn: Electtaria cardamom*	Zingiberaceae		CL	1
***90***	Hel	Cardamom	Ḥamāmā	*Amomum cardamomum*	Zingiberaceae	/ Decoction	OL, LD	2
***91***	Zanje(a)bil	Dried ginger	Zanjabil	*Zingiber officinale*	Zingiberaceae		CL	1
***92***	???					/ Cooked	PL	1
***93***	Kāsni biyābāni	Wild endive	Ṭarakhshaqūq			/ Extract	D, OL	2

Abbreviations: J: Jaundice; BH: Remove bilious humours, expels bilious humours; OL: Removes the obstructions of liver, removes hepatic obstructions, de obstruent for the liver; CLL: Cleanses the liver, cleanses (the foul humours of) the liver; PL: Pain of the liver, hepatalgia, painful conditions of liver; PF: Prevents the flow of bile towards the viscera, prevents the infiltration of yellow bile towards the intestines; BN: Bilious Nausea; CL: Strengthens 'cold' liver, 'coldness' (atony) of the liver, suitable for the 'coldness' of the liver; FD: Facilitates downward flow of bile in the urinary tract; D: Dropsy (Ascites); RY: Removes 'yellow water' (ascetic fluid); GL: Good for liver; HBF: Head bile filtration; EXB: Expels yellow bile; UL: useful for the liver (ailments), beneficial for the liver, helpful to the liver; EXC: Counteracts the ill effects of excessive yellow bile; LT: Useful for the liver Temperament; BD: Useful in bile diarrhea, bilious diarrhea, stop bilious diarrhea; EYB: Eradicating the yellow bile; STL: strengthens the liver; CB: Checks yellow bile; PB: Purged out bile, purges out the 'burnt' bile; GIL: gaseous inflation of the liver; VB: vomiting of bile, stops biliary vomiting, bilious vomiting; EVY: Evacuates the yellow bile, evacuates the burnt bile; PH: Produces heat in liver, warming drug for liver; IL: Inflammatory conditions of the liver; EBB: Evacuates the black bile; I: Itch, urticarial, prurigo, scabies; LD: Liver disease; ED: Expels (dropsical) water; AL: Atony of the liver; OJ: Obstructive jaundice; LI: Liver injuries caused by fall, damaged liver; BT: Bilious thirst; SIN: stops infiltration of yellow bile towards stomach; RB: Removes yellow bile; IRL: Irritation of liver; EY: Eliminates yellow bile; PE: Prevents excessive secretions; REB: Relieves bile; CM: Controls the 'movement' of yellow bile; EJ: Eye jaundice.

**Table 2:** Properties of herbs that were found effective against spleen disease, mentioned in the 2nd volume of Canon

***No.***	***Persian name***	***Common name***	***Arabic name***	***Scientific Name***	***Family***	***Used Parts/*** ***Mode of consumption or preparation***	***Conditions which the herb has effect on***	***Score***
***1***	Karafs koohi	Rock-parsley, southern wood	Būyānas	*Petroselinum sativum *Hoffm, nom. nud.	Apiaceae		SS	1
***2***	Čātlānquš, saqqez, bane	Terebinth	Ḥabba al-khaḍrā,' botm	*Pistacia terebinthus *L.	Anacardiaceae	Resin, gum	SD, I	2
***3***	Ašaqe'	Labdanum	Qissūs, gheysus	*Hedera helix *L*. *	Araliaceae	Fresh / plastering	US	1
***4***	Kabar	Caper, caprifole	Kabar	*Capparis spinosa *L.	Capparidaceae	Root-bark / orally or plaster	HS, EM	2
***5***	Hezār gušān, fāserā, tāk e sefid	White bryoni	Fāshrā, karma baiḍā,' hazārjashān	*Bryonia alba *L.	Cucurbitaceae		GS	1
***6***	Nil	True indigo	Nil	*Indigofera linifolia *(L.f.) Retz*.*	Fabaceae	Wild variety	S	1
***7***	Bi'al	Alfalfa	Abi'al	*Medicago sativa *L*.*	Fabaceae		US	1
***8***	Šamba(e)lile	Fenugreek	Ḥulbah,	*Trigonella foenum-graecum *L*.*	Fabaceae	/ painted	US	1
***9***	Ezār če(a)šm	Hypericon	Hiōfāriqūn, 'arn, 'inab al-hayyah	*Hypericum perforatum*	Hypericaceae	Fruits	PBB	1
***10***	Tarfondos, tarfooless	Teukrion	Ṭūqriūs ṭarqoyūs, ṭarfūlis	*Teucrium flanum *L*.*	Lamiaceae	Pieces / decoction, plastered	SSW, HS	2
***11***	Gole arbe	Cat thyme, hulwort, mountain germander	Jo ͨdah	*Teucrium polium *L.	Lamiaceae	/ painted, used with vinegar, decoction of large variety	S, HS, BJ, D	4
***12***	Nilōfar, kalam e ābi'	Water lily, sea-kale	Nilōfar, hab[b] ol arus'	*Nymphaea lotus *L*.*	Nymphaeaceae	Root / orally or plaster,	S	1
***13***	Felfel	Pepper	Filfil	*Piper sp.*	Piperaceae	/ orally or painted	SI	1
***14***	Šaytarak	Lepidium	Shitaraj	*Plumbago zeylanica *L*.*	Plumbaginaceae	/ paint	SHS	2
***15***	Gazmāzu, gazmāzak	Tamarisk nut	Jauz al-ṭarfā, kazmārak, asl,' ṭarfā	*Tamarix gallica *L*.*	Tamaricaceae	Branches, leaves / decoction, plastered	SD, HS,	2
***16***	Dāruš	Mistletoe	Dibq	*Viscum album *L*.*	Viscaceae	/ poultice	I, SSW	2

Abbreviations: SS: sclerosis of spleen; SD: Splenic disease, splenic disorders; I: Itch, urticarial, prurigo, scabies; US: useful for spleen, useful in splenic ailments; PBB: Purge out black bile, HS: Hardness of the spleen, splenic hardness; EM: Evacuates the thick melanotic matters of the spleen; GS: Good for spleen, good drugs for the spleen; S: Splenitis; SSW: splenic swelling; BJ: Useful in black jaundice, melanotic jaundice; D: Dropsy (Ascites); SI: Spleen inflammation; SHS: shrinks the (enlarged) spleen, emaciates the spleen, splenic enlargement, reduces the size of spleen.

**Table 3:** Plants used as medicinal agent in liver and spleen

***No.***	***Persian name***	***Common name***	***Arabic name***	***Scientific Name***	***Family***	***Used Parts/*** ***Mode of consumption or preparation***	***Conditions which the herb has effect on***	***Score***
***1***	Agar'	Sweet scented flag	Waj	*Acorus calamus *L.	Acoraceae		CH, STL, HS, SHS	4
***2***	Falanje	Leek	Ḥirbah	*Allium porrum* *Syn, Allium ampeloprasum*	Amaryllidaceae	Peels, leaves / dried, orally,	SD, H	2
***3***	Mastaki	Mastic, mastiche tree gum	Maṣṭaki	*Pistacia lentiscus *L.	Anacardiaceae	Roots / taken orally, plaster	S, STL, LD, LW, IL	4
***4***	Karafs	Ceiery	Karafs	*Apium graveolens *L.	Apiaceae	Seed / orally	GL,GS, D, TL, CL	5
***5***	Oše'		Ushaq, lazaq al zahab, ṭarthoth	*Dorema ammoniacum *(D.Don) *Syn: Gum ammoniac* *Syn, Ferula ammoniacum*	Apiaceae	/ used internally, painted	HS, LH, D	3
***6***	Gāvšir	Opopanax, galbanum	Jāoshir	*Opopanax chironoum, *(L.) Koch.	Apiaceae	/ juice, with vinegar,	HS, S, D	3
***7***	Anisūn, rāziyāne rumi	Anise, anis	Anisūn	*Pimpinella anisum*	Apiaceae		OL, OS	2
***8***	Barbāle	Indian valerian	Asārūn	*Asarum europaeum *L*.*	Aristolochiaceae	/ Infusion (naqi')	D, OL, LH, HS, J, GD	6
***9***	Sarāvand	Zarawand, Indian birthwort	Zarāwand, arestolokhia	*Aristolochia longa *L*.* *Syn, Aristolochia fontanesii*	Aristolochiaceae	round variety / with oxymel, painted, powdered, orally	SD, GS, PB	3
***10***	Zangidāru	Spleen wort	Saqūlūqandriūn, kaf al-nasr	*Asplenium scolopendrium *L.	Aspleniaceae	Leaves / decocted	GS, SD, J	3
***11***	Afsantin, de(a)rmane rumi	Absinth, absinthe, worm wood	Afsantin	*Artimisia absinthium *L*.*	Asteraceae	/ syrup, extract, plaster, ointment, pessary	J, D, US, PL, LH, PB, BH	7
***12***	Moškāniyye	Hemp agrimony	Ghāfith	*Eupatorium cannabinum*	Asteraceae	/ oral intake, extract	I, PL, OL, STL, LH, H, D	7
***13***	Bābune gāv če(a)šm, amārion, arqasmun, qurinbun	Bachelo's buttons	Uqhuwān	*Pyrethrum parthenium* *Syn, Tanacetum parthenium*	Asteraceae	/ oil	PBB, SS	2
***14***	Šire ye. Fil zahre	Extract of ophthalmic berberry	Ḥoḍaḍ hendi	*Berberis aristata *DC	Berberidaceae	/ oral intake or external painting of indian variety	SD, BJ	2
***15***	Gole qāsed	Shanjar, dyer's bugloss	Shanjār, khas al-ḥimār	*Alkanna sp.*	Boraginaceae	/ rub anāqalyā variety, oral intake, plaster, preserved in vinega	J, SA, CLL, SD	4
***16***	Hovečube	Dyers bugloss	Abū halsā, abū khalsā, shenjār	*Anchusa tinctoria *L.	Boraginaceae	/ decoction	J, SA, BH	3
***17***	Kalam	Cabbage	Kurunb	*Brassica oleracea*	Brassicaceae	Leaves / extract	J, SD	2
***18***	Barqast	Asclepias	Qunna barā	*Lepidium draba *L.	Brassicaceae		OL, OS	2
***19***	Toxm taretizak biyābāni, šāhtare	Garden cress	Ḥurf	*Lepidium sativum *L. *Nasturtium officinale *R.brr	Brassicaceae	Babylonian cress / plaster	PH, SHS, VB, EL	4
***20***	Tor[o]b	Radish	Fujl	*Raphanus sativus *L.	Brassicaceae	Seed, leaves / plastered, extract,	GS, SI, OL, J, PL, D	6
***21***	Sonbol, sonbole hendi	Nard, indian spikenard	Sunbul	*Nardostachys jatamansi *D.C*.*	Caprifoliaceae		OL, STL, J, US	4
***22***	Palaxam	Struthion	Kundus	*Gypsophila struthium *L.	Caryophyllaceae		DB, HS	2
***23***	Selq	Beet	Silq	*Beta vulgaris *L.	Chenopidiaceae		OL, S	2
***24***	Halile	Chebulic myrobalan	Halilaj	*Terminalia chebula*	Combretaceae	Kābuli variety, yellow variety Black variety	SA, UB D, EBB EVY EBB	5
***25***	Lablāb	Lablab	Lablāb	*Convolvulus arvensis *L.	Convolvulaceae	Leaves / juice	OL, S, PB	3
***26***	Faqilāsus, bo(e)xor maryam	Faqlaminus	Faqlāminūs, bukhūr maryam	*Cucumis sativus *L.	Cucurbitaceae	/ oral intake, extract, plastered	J, S	2
***27***	Noxod	Gram, chickpea	Ḥimmaṣ	*Cicer arietinum *L.	Fabaceae	/ flour, decoction, coloured and black varieties	D, J, OL, OS	4
***28***	Lubiyā gorgi	Lupine	Turmus	*Lupinus albus *L.	Fabaceae	/ cooked	OL, OS	2
***29***	Gole gandom	Common centaury	Qanṭūriūn, luqaye koochak, lambison, qanṭūriūn saqir	*Centaurium erythraea *Rafn	Gentianaceae	/ decoction	OL, HS, PB	3
***30***	Je(a)ntiyānā	Gentian	Janṭiānā, kaf foz ze'b	*Gentiana lutea *L*.*	Gentianaceae	/ taken with wine	OL, OS, PL, SA, CL, SSW	6
***31***	Ishqil	Squill	Ishqil	*Urginea Indica * *Syn, Drimia indica*	Hyacinthaceae	/ it's viniger, decoction, kept hanging on the body	SS, DI, SHS, D, J	5
***32***	Za'fe(a)rān	Safron	Za'farān	*Crocus sativus *L*.*	Iridaceae		STL, GS	2
***33***	Susan	Lily	Sausan, iris, sausan el āsemajooni, irsā	*Iris florentina *L.	Iridaceae	/ oil	S, BI	2
***34***	Zambaq, iris	Orris root	Irsā, sowsan	*Iris ensata*	Iridaceae	/ with vinegar, internal and external use, old powdered,	CH, CS, D, EXB, EB	7
***35***	Māš dāru	Tecrium	Kamāfiṭūs, khamāfitūs	*Ajuga chamaepitys *(L.) Schreb	Lamiaceae		LD, OL, SD, MJ	4
***36***	Hesl, zufā ye xošk	Hyssop	Zūfa yābis, zavān	*Hyssopus officinalis *L*.*	Lamiaceae	/ plastered, oral intake	GS, D	2
***37***	Gandnā ye koohi	Black horehound	Farāsiūn' , alqam	*Marrubium vulgare *L.	Lamiaceae		OL, OS	2
***38***	Pune	Mint	Fūdhanj	*Mentha piperita *L. *Syn. Mentha x piperita*	Lamiaceae	wild variety, mountain mint / decoction, bath, ointment, plastered, orally	BC, BO, BL, J, D, SHS, RBB	7
***39***	Maryam noxodi	Common germander	Kamādriūs, kamāzriūs	*Teucrium chamaedrys* L*.*	Lamiaceae	/ old	HS, MJ, D	3
***40***	Panj[']angošt	Chaste tree	Banjanjusht	*Vitex agnus-castus*	Lamiaceae		OL, OS, SS, D	4
***41***	Dahmašt,	Bay tree, seed of laurel	Dahmusht, qār, ḥab al-ghār	*Laurus nobilis *L*.*	Lauraceae	Oil, peel,	H, S, PL	3
***42***	Anjir'	Fig, fig tree	Tin	*Ficus carica *L.	Moraceae	cluster fig, leaves / decoction, juice	OL, OS, D, SS, I,	5
***43***	Hab[b] al bān	Persian lilac	Bān	*Moringa arborea *Verdcourt	Moringaceae	Fruit / plaster	SL, SS, US	3
***44***	Čārgun, bazbāz, basbāse	Mace, nutmeg	Bisbāsah, jauz būwwā	*Myristica fragrans *Houf*.*	Myristicaceae	Nut	STL, STS	2
***45***	Baspāyak	Common polypody	Bisbāij	*Polypodium vulgare *L*.*	Polypodiaceae	Root / powdered	PBB, PB	2
***46***	Qārč e deraxti	White agaric	Ghāriqūn	*Polyporus officinalis*	Polyporaceae		J, SI, PL	3
***47***	Toršak	Yellow dock, sour dock	Ḥummāḍ	*Rumex crispus *L*.*	Polygonaceae	/ decoction, bath with its water, with wine, cooked with vinegar and plastered	RB, I, BJ, SSW	4
***48***	Rivand, rivand.e čini, behman, rāvand	Himalayan rhubarb	Riwand	*Rheum officinale *L. *Rheum emodi *Wall. ex Meisn.	Polygonaceae		LD, PL, LW, SHS	4
***49***	Parsiyāvo(a)šān	Maiden hair	Barshiāushān	*Adiantum capillus veneris *L.	Pteridaceae	/ administration with wine	SA, J	2
***50***	Bādām	Almond	Lauz	*Amygdalus communis *L.	Rosaceae	Seed /oil, flour	OL, OS, GS, SU, S	5
***51***	Ru(o)nās	Dyers madder	Fūwah al-ṣabbāghin	*Rubia tinctorum*	Rubiaceae	Fruit	S, CLL, CLS, OL, OS	5
***52***	Fayjan	Common rue	Sozāb, sodāb	*Ruta graveolens *L*.*	Rutaceae	/ plastered, decoction	D, GS	2
***53***	Filzahre		Filzahraj	*Lycium afrum *L *Rhamnus saxatilis *L.	Solanaceae Rhamnaceae	Branch / decocted, orally	S, J	2
***54***	Haftbarg	Mazerion	Mādhriūm	*Daphne mezereum *L.	Thymelaeaceae	/ Electuary, suppository,	D, BD, PBB	3

Abbreviations: CH: Cold hepatalgia; STL: strengthens the liver; HS: Hardness of the spleen, splenic hardness; SHS: shrinks the (enlarged) spleen, emaciates the spleen, splenic enlargement, reduces the size of spleen; SD: Splenic disease, splenic disorders; H: Hepatitis; S: Splenitis; LD: Liver disease; LW: Liver weakness; IL: Inflammatory conditions of the liver; GL: Good for liver; GS: Good for spleen, good drugs for the spleen; D: Dropsy (Ascites); TL: tones up the liver; CL: Strengthens 'cold' liver, 'coldness' (atony) of the liver, suitable for the 'coldness' of the liver; LH: Liver hardness; OL: Removes the obstructions of liver, removes hepatic obstructions, de obstruent for the liver; OS: Obstructions of spleen, de obstruent for the spleen; J: Jaundice; GD: General Dropsy (anasarca); PB: Purged out bile, purges out the 'burnt' bile; US: useful for spleen, useful in splenic ailments; PL: Pain of the liver, hepatalgia, painful conditions of liver; BH: Remove bilious humours, expels bilious humours; I: Itch, urticarial, prurigo, scabies; PBB: Purge out black bile; SS: sclerosis of spleen; BJ: Useful in black jaundice, melanotic jaundice; SA: Splenalgia, splenic pain; CLL: Cleanses the liver, cleanses (the foul humours of) the liver; PH: Produces heat in liver, warming drug for liver; VB: vomiting of bile, stops biliary vomiting, bilious vomiting; EL: Expels bile through loose motion; SI: Spleen inflammation; DB: Diluent black bile; UB: useful for bile ailments; EBB: Evacuates the black bile; EVY: Evacuates the yellow bile, evacuates the burnt bile; SSW: splenic swelling; DI: Dissolves inflammation of the spleen; BI: Biliary ileus; CS: Cold spleenalgia; EXB: Expels yellow bile; EB: Expels the black bile; MJ: Melanotic jaundice; BC: Bile clean up; BO: Bile opener; BL: Bile laxative; RBB: Remove black bile; SL: Sclerosis of liver; STS: strengthens the spleen; RB: Removes yellow bile; SU: stirs up yellow bile; CLS: Cleanses the spleen; BD: Useful in bile diarrhea, bilious diarrhea, stop bilious diarrhea.

The most common effective plants prescribed for liver or spleen diseases or both, are shown in Figure 2. Figure 3 shows the plant families (i.e. Lamiaceae, Fabaceae, Apiaceae, Rosaceae, Asteraceae, Solanaceae, and Brassicaceae) with the largest contribution to development of treatments against liver and spleen diseases.

**Animals**

The majority of the 17 agents of animal’s origin including animal, organs or animal physiological fluids used as medication. In certain cases, especially for insects, the whole body was used, and in other cases, the animal’s urine or even the milk was used. The animals and their applications are presented in Table 4.

The used animals can be divided into groups according to their availability, such as domesticated animals, such as jennet, goat, camel, cow, and others such as locust and worms. Wild animals, for example, wolf, sand grouse, red-headed partridge, hedgehog, porcupine, antelope, and stag. Various organs, and products of exotic animals, species such as Spanish fly, coral, oyster have been used in order to cure liver and spleen diseases

**Table 4:** Animals and their parts used as a source of medication

***No.***	***Persian name***	***Common name***	***Arabic name***	***Scientific Name***	***Family***	***Used Parts/*** ***Mode of consumption or preparation***	***Diseases Which the agent has Effect on***	***Score***
***1***	Badal e marjān	Coral	Bussad	*Corallium rubrum*	Coralliidae	/ mixed with water	SSW	1
***2***	'Edrār	Urine	Baul	*Peri ouron*		Drink / Human, camel Human	I D, HS J, SD	5
***3***	Malax	Locust	Jarād	*Locusta migratoria *	Acridida		D	1
***4***	Kerm	Worm	Dūd, *d*ū*d al-ṣabbāghin*			/ the red multi legged worms with wine	J	1
***5***	Kaf e daryā	Sea foam, casting of king fisher	Zabad al-baḥr	*Alcyonium*	Alcyoniidae	Rosy kind	SD, D	2
***6***	Jegar	Liver	Kabid	*Hepar*	Wolf liver		PL	1
***7***	Šayyer	Milk	Laban	*Gala*	Jennet Goat Camel Cow	Milk Cheese water, Doogh, Boiled sour milk	I, J J D, HS HS, HS, SD, LD, SSW, IL, GL, D EXB BD	11
***8***	Gušt	Meat	Laḥm		Sand grouse Hedgehog Cow		D, OL, OS, LW D PF, BD	6
***9***	'Madfu	Faeces, excreta, stool	zibl	*Dung*	Goat, mountain goats Goat Human	Oral intake / plaster or paint, taken with some aromatics	J, HS D J	3
***10***	'Osto(e)xān	Bones	ͨiẓām	*Os*	He-goat	Oral intake of ankle bone	SSW	1
***11***	Sadaf	Pearl, oyster shell			Farofas Oyster	/ poultice	SD, D D	
***12***	Šāx e jānevarān	Horn	Qarn	*Cornu*	Stag	/ washed and burnt	D, J	2
***13***	Sang xārak	Sand grouse	Qaṭā				D, Pbb	2
***14***	Xārpošt e biyābāni	Porcupine	Qunfudh barri	*Hystrix cristata*		Flesh/ salted Liver / sun dried	D	1
***15***	Kabk	Red headed partridge	Qabaj, ghag			Meat	D	1
***16***	Kerm e sorx	Earth worm	Kharātin	*Lumbricus*	Lumbricidae	/ orally	J	1
***17***	Āl[l]ākolang'	Spanish fly	Dharāriḥ	*Cantharidus vesicatoria*	Trochidae		D	1

Abbreviations: SSW: splenic swelling; I: Itch, urticarial, prurigo, scabies; D: Dropsy (Ascites); HS: Hardness of the spleen, splenic hardness; J: Jaundice; SD: Splenic disease, splenic disorders; PL: Pain of the liver, hepatalgia, painful conditions of liver; LD: Liver disease; IL: Inflammatory conditions of the liver; GL: Good for liver; EXB: Expels yellow bile; BD: Useful in bile diarrhea, bilious diarrhea, stop bilious diarrhea; OL: Removes the obstructions of liver, removes hepatic obstructions, de obstruent for the liver; OS: Obstructions of spleen, de obstruent for the spleen; LW: Liver weakness; PF: Prevents the flow of bile towards the viscera, prevents the infiltration of yellow bile towards the intestines; Pbb: Produces black bile.

**Table 5:** Minerals noted in Canon by Avicenna

***No.***	***Persian name***	***Common name***	***Arabic name***	***Scientific Name***	***Used Parts/*** ***Mode of consumption or preparation***	***Diseases Which the agent has Effect on***	***Score***
***1***	Mum[i]yā	Asphat, mineral pitch, jews pitch	Mūmiāi	*Asphaltum*	Snuff, oral use	LI, SA	2
***2***	Namak e čini, gel e Āsious		Asyūs	*Asian stone*	/ Painting	US	1
***3***	Burak	Borax	Būraq	*Natron*	/ Plaster	D, I	2
***4***	Āhan'	Iron	Ḥadid	*Ferrum*	Extinguished hot iron in wine and water	S	1
***5***	Gel e ma'muli	Common earth	Ṭin muṭlaq		from a sunny land / Painted	D, S, GD	3
***6***	Gel e maqarra	Red ochre	Maghra	*Bolus armenus rubra*		PL	1
***7***	Āb'	Water	Mā'	*Aqua*	Iron rich water, Copper containing water Sea-water Sea- water / vapours hot spring water, Sulphuric water	US I D IL, PL, SI, SA	7
***8***	Namak	Salt	Milḥ	*Sodium chloride*	/ Paint Nifṭi salt,	I BB	2

Abbreviations: LI: Liver injuries caused by fall, damaged liver; SA: Splenalgia, splenic pain; US: useful for spleen, useful in splenic ailments; D: Dropsy (Ascites); I: Itch, urticarial, prurigo, scabies; S: Splenitis; GD: General Dropsy (anasarca); PL: Pain of the liver, hepatalgia, painful conditions of liver; IL: Inflammatory conditions of the liver; SI: Spleen inflammation; BB: Bile break.

**Table 6:** Processed agent used in medicine in canon in medicine medication by Avicenna

***No.***	***Persian name***	***Common name***	***Arabic name***	***Scientific Name***	***Used Parts/*** ***Mode of consumption or preparation***	***Diseases Which the agent has Effect on***	***Score***
***1***	Serke	Vinegar	Khal	*Acetum vinegar*	/ Fumigation	D	1
***2***	Nešāste	Starch	Nashā		/ Oral use	BD	1
***3***	La'l e moa'bberi	A resin	Qaiqahan, qanqahar	*Qaiqahan*	/ Orally	SHS	1
***4***	Panir	Cheese	Jubn	*Serparium*	The water of cheese	PBH, EXB	2
***5***	Omaali, ormaali, asal' e dāvud, o[w]qan e asal'	Honey wine or mead	Ūmāli	*Eleemali muslum*	/ Diluted with water	PB	1

Abbreviations: D: Dropsy (Ascites); BD: Useful in bile diarrhea, bilious diarrhea, stop bilious diarrhea; SHS: shrinks the (enlarged) spleen, emaciates the spleen, splenic enlargement, reduces the size of spleen; PBH: Produces bilious humours; EXB: Expels yellow bile; PB: Purged out bile, purges out the 'burnt' bile.

**Minerals**

We were able to identify 8 materials of mineral origin (Table 5). The use of such agents in medicine has been well-known throughout history. 

**Agents of other origins**

Five medicinal agents that were processed from animal or plant materials or agents of mixed or unknown origin are presented in Table 6. 

**Table 7:** Effective Medicinal agent for liver and spleen diseases grouped based on their origin

***Origin ***	***Number***	***Percentage***
***Plants***	163	84.455
***Animals***	17	8.81
***Minerals***	8	4.145
***Processed***	5	2.59
***Sum***	193	100

## Discussion

The overwhelming majority (84.45%) of agents that served as simple drugs were derived only from plants (see Table 7). The proportion of materials derived from animals and animal organs is small (8.80%), and minerals represent an even smaller proportion (4.14%). 

Based on our survey of Canon, 163 herbal parts which exert therapeutic effects on the liver and spleen, were found. In this book, some items refer to one herb Ṭarfūlis and Ṭūqriūs both refer to Teukrion or some items are parts of one herb and have different names e.g. Ḥiltit is gum and Maḥrūth is the root of Anjudhān so the last two have same scientific name of Asafoetida in the Tables. 

In old manuscripts, there are different descriptions for identical herb so different scientific names have been proposed for the same herb. Prof. Ghahraman and Prof. Okhovvat have introduced appropriate scientific names for old names; in the present study, we used scientific names according to their suggestions (Ghahreman and Okhovvat, 2004 ▶).

From antiquity until now herbalist and medicinal experts had a quest to find the most effective herb that has the richest source of medicinal material, in order to use it in liver tonics and other formulas. Our research revealed that the effective herbs are not limited to one family but the most frequently used herbs belong to several families that are given in Figure 3. Some families like Laminacea have higher numbers of herbs that are frequently used as effective therapeutics for spleen and liver diseases. We propose to study the herbs of this family and other families noted in Figure 2 in order to find the most effective herb for treatment of liver and spleen diseases.

According to the number of effects that every herb has, as prescribed by Avicenna as effective on liver or spleen diseases or both, the herbs were scored (Figure 2). Ghāfith (*Eupatorium cannabinum*) was named "The eupatorion of Avicenna" (Tobyn et al., 2016 ▶) in old times, is one of the highly scored herbs. The present studies demonstrated choleretic and hepatoprotective effects of hemp agrimony (Lexa et al., 1989 ▶) although it contains pyrrolizidine alkaloids (Edgar et al., 1992 ▶;Hendriks et al., 1987 ▶) which have hepatotoxic and potentially carcinogenic and genotoxic effects and essential oils of *E. cannabinum* is notably toxic (Judzentiene et al., 2016 ▶). On the other hand, the effects of a plant such as Irsā (*Iris ensata*) which has the highest score (Figure 1), on the liver or spleen, have not yet been reported and its medicinal use had been uncertain or unknown according to lack of articles is considered a good candidate for future investigations. Fūdhanj (*Mentha piperita*) which also gained a high score was used successfully by Avicenna as a drug, for treatment of liver and spleen diseases. It was shown that *M. piperita* causes lipid peroxidation and hepatic damage in a dose-dependent manner (Akdogan et al., 2004 ▶). It has hepatotoxic potential (Douros et al., 2016 ▶) and moderately severe adverse effects (Posadzki et al., 2013 ▶), further studies in this field are needed. Meanwhile, *M. piperita *has radioprotective properties against gamma irradiation which is probably mediated via its antioxidant and free radical scavenging activities of leaf extract (Samarth et al., 2006 ▶); also, this plant may be useful for reducing the side effects of arsenic-induced hepatopathy (Sharma et al., 2007 ▶). Afsantin* (Artimisia absinthium*) is another high-score herb which exhibits hepatoprotective action partly through microsomal drug metabolizing enzymes (MDME) inhibitory action (Gilani and Janbaz, 1995 ▶), has significant antioxidant activity and protects the liver and kidney (Kharoubi et al., 2008 ▶) probably through its immunomodulatory activity (Amat et al., 2010 ▶). Also, this plant was considered for reducing hepatic damage and it may serve as an alternative medicine in hepatic conditions (Saxena and Shukla, 2012 ▶). 

These results show a need for a close scrutiny in methods of planting, harvesting, processing, extraction and preparing a single or combination formula that affects remedies and practice of ancient medicine. In order to prepare a suitable herbal drug for the treatment of complicated diseases such as liver cirrhosis and hepatocellular carcinoma, according to Avicenna book, further studies are needed to make an effective drug for liver or spleen diseases is promising.

Through analysis of 806 therapeutic items of Avicenna 2^nd^ volume of Canon, we prepared a list of agents that are effective in three main areas namely, liver, spleen, and liver & spleen diseases. The current study indicates the necessity of deep analysis, study and further assessment of listed items.
